# Digital health, microfluidics, and bedside genetic testing

**DOI:** 10.1186/1755-8166-7-S1-I55

**Published:** 2014-01-21

**Authors:** Syed Hashsham

**Affiliations:** 1 Department of Civil and Environmental Engineering, Michigan State University, USA

## 

Decentralized, bedside, or point of care analysis of nucleic acids (DNA/RNA/mRNA/microRNA)-based markers is expected to be a key component of digital healthcare. Nucleic acids-based approaches allow for screening of disease and pathogens, disease surveillance, selection of treatment, treatment effectiveness, differential diagnosis, risk assessment, staging, and prognosis. Hand-held genetic analysis systems have the potential to provide all these capabilities in a simple, affordable, field deployable, rapid, multiplexed, and robust manner without the need for electrical power or refrigerated reagents. Even the need for specific language can be eliminated by translatable or visual graphical user interface truly integrating the analytical system with the communication network.

The presentation will introduce Gene-Z and iDx, two networkable platforms that are developed to provide simplified analysis of genetic markers. Gene-Z has Android/iPod based operation using Bluetooth^TM^ and capable of carrying out quantitative isothermal amplification for 64 reactions in a disposable microfluidic chip. The device is battery operated and can be charged by solar panels integrated at the top of the device. A smaller version (iDx) working as an attachment to cell phones, also with real time amplification and quantification capabilities, allows 8 reactions in parallel. Both these devices are networkable and currently being validated for a number of key applications important to human and animal health, plant safety, industrial microbiology, and environmental protection. Simplification, reliability, and cost-effectiveness are key factors ensuring successful implementation of such approaches. Business model for adoption of such low cost approaches, however, is still evolving and major challenges need to be addressed about how to sustain a business that is built on small margins usually by small companies engaged in biomedical research.

